# Dynamics of the STAT3 Transcription Factor: Nuclear Import Dependent on Ran and Importin-β1

**DOI:** 10.1371/journal.pone.0020188

**Published:** 2011-05-19

**Authors:** Velasco Cimica, Hui-Chen Chen, Janaki K. Iyer, Nancy C. Reich

**Affiliations:** Department of Molecular Genetics and Microbiology, Stony Brook University, Stony Brook, New York, United States of America; Cleveland Clinic, United States of America

## Abstract

The signal transducer and activator of transcription-3 (STAT3) induces transcription of genes that control differentiation, inflammation, proliferation, and tumor cell invasion. Cytokines such as interleukin-6 and interferon stimulate the specific tyrosine phosphorylation of STAT3, which confers its ability to bind consensus DNA targets. In addition, unphosphorylated STAT3 has been demonstrated to induce specific gene expression. STAT3 must gain entrance to the nucleus to impact transcription, however access to the nucleus is a tightly regulated process. Because nuclear trafficking is critical to the function of STAT3, we investigated the molecular mechanisms by which STAT3 is imported to the nucleus. Live cell imaging techniques were used with STAT3 tagged with green fluorescence protein (GFP) or photoactivatable GFP to follow the cellular dynamics of both unphosphorylated and tyrosine phosphorylated forms. Cytokine activation did not alter the rate of STAT3 nuclear import or nuclear export. In addition, Förster resonance energy transfer experiments revealed homomeric interaction of unphosphorylated STAT3 dependent on its amino terminus, but this dimerization is not necessary for its nuclear import. Previous work demonstrated the adapter importin-α3 binds to STAT3 and is required for nuclear import. To determine whether STAT3 nuclear import is mediated by the importin-α/importin-β1 heterodimer, the effects of siRNA to importin-β1 were evaluated. Results indicate STAT3 nuclear import is dependent on the function of importin-β1. Since the Ran GTPase is necessary to bind importin-β1 in the nucleus for release of importin-α-cargo, the effect of a GTPase deficient mutant of Ran was tested. Expression of the Ran interfering mutant inhibited STAT3 nuclear import. This study defines importin-α/importin-β1/Ran as the molecular mechanism by which STAT3 traffics to the nucleus.

## Introduction

The biological functions of the signal transducer and activator of transcription 3 (STAT3) are diverse. Deletion of the STAT3 gene in mice results in embryonic lethality, and studies with conditional gene targeting have revealed impaired differentiation, proliferation, migration, or apoptosis in a wide variety of tissues [1], [2], [3]. In contrast to deficiency, persistent activity of STAT3 plays a causative role in the development of life-threatening diseases that include cancer and autoimmunity [4], [5], [6]. STAT3 was first identified as a DNA binding factor activated by the interleukin-6 (IL-6) cytokine, but it is now known to be activated by a number of cytokines, growth factors, and oncogenic tyrosine kinases [7], [8], [9], [10]. Classical activation of STAT3 occurs with tyrosine phosphorylation by Janus kinases (JAKs) in response to cytokine binding to cell surface receptors [10], [11]. Phosphorylation of tyrosine 705 promotes dimerization through reciprocal interactions of phosphotyrosine and Src homology 2 (SH2) domains in STAT3 monomers. This dimer conformation enables STAT3 to bind to consensus DNA targets and induce gene expression. Recent studies also have provided evidence for noncanonical mechanisms of STAT3 function that are independent of tyrosine phosphorylation or DNA binding [12], [13], [14], [15], [16]. For example, unphosphorylated STAT3 can stimulate expression of pro-inflammatory and pro-oncogenic genes either independent or dependent on binding to nuclear factor-κB [13]. Therefore irrespective of its state of tyrosine phosphorylation, nuclear trafficking is a key regulatory mechanism of STAT3 transcriptional activity.

Access to the cellular genome is limited by passage through nuclear pore complexes (NPC) within the nuclear membrane [17]. Small molecules can diffuse through the NPC, but transport of large proteins is restricted to those that possess a nuclear localization signal (NLS) or nuclear export signal (NES) [18], [19], [20], [21]. The signal sequences are recognized directly or indirectly by the karyopherin-β family of proteins (importins and exportins) that interact with the NPC and facilitate transport. Importin-β1 commonly binds cargo indirectly through adapter molecules of the importin-α family. Importin-α adapters bind directly to the NLS in cargo, and also bind to importin-β1. Our previous studies demonstrated the ability of the specific importin-α3 adapter to bind STAT3 [22]. Importin-β1 mediates import of the importin-α-NLS cargo through the NPC.

The direction of protein transport into or out of the nucleus is controlled by a gradient of Ran-GTP [23], [24]. Ran is a small GTPase found in two nucleotide forms bound to GTP or GDP. Nucleotide exchange factors in the nucleus and GTPase activating proteins in the cytoplasm create a relatively high ratio of Ran-GTP to Ran-GDP in the nucleus, and an inverse ratio in the cytoplasm. Following entrance to the nucleus, importin-β1 binds to Ran-GTP causing a conformational change and release of the importin-α-NLS cargo [25]. The nuclear export process is also regulated by high levels of Ran-GTP in the nucleus and low levels in the cytoplasm. Proteins that are destined for export form a stable ternary complex with an exportin transport factor and Ran-GTP. Following transport to the cytoplasm, stimulation of Ran GTPase leads to release of cargo.

Regulation of transcription factor access to the nucleus provides a means to turn on or to turn off specific gene expression. Nuclear trafficking of STAT3 is therefore pivotal to its function as a transcription factor. We have demonstrated previously that nuclear import of STAT3 is independent of its tyrosine phosphorylation, and provided evidence for the requirement of importin-α3 [22], [26]. However the mechanisms that regulate STAT3 nuclear import still remain unsettled [27], [28], [29], [30], [31]. To address this critical issue we applied techniques of live cell imaging. Photobleaching and photoactivation techniques were used to study the movement of STAT3 in and out of the nucleus in real time, and Förster resonance energy transfer was used to evaluate STAT3 protein interactions. These techniques were combined with genetic tools to evaluate the contribution of importin-β1 to the nuclear import of STAT3. Our results demonstrate the nuclear import of STAT3 is independent of tyrosine phosphorylation, but is dependent on the action of Ran GTPase and importin-β1. The studies indicate that the STAT3 protein possesses a constitutive nuclear localization signal and its nuclear import is mediated by the importin-α-importin-β1 heterodimer pathway. Knowledge of the mechanisms that regulate STAT3 nuclear trafficking is essential to develop strategies for its intervention.

## Results

### Live cell imaging captures STAT3-GFP localization in the nucleus

**Figure 1:**
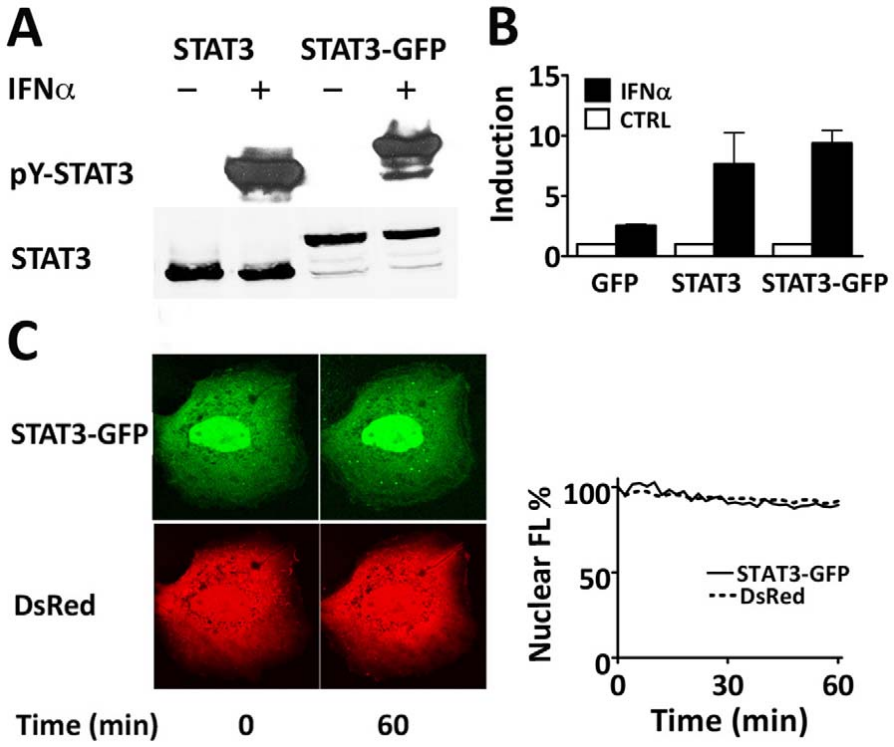
Prominent nuclear localization of STAT3-GFP independent of tyrosine phosphorylation. **A**) Western blot of cell lysates demonstrate tyrosine phosphorylation of STAT3 and STAT3-GFP in response to cytokine. 293 cells were transfected with untagged STAT3 or STAT3-GFP expression plasmids, serum-starved, and either untreated or treated with interferon-α (IFNα) for one hour. Separate blots using antibodies to STAT3 or to the specific phosphorylated tyrosine 705 of STAT3 (p-Y-STAT) are shown **B**) Induction of a GAS-luciferase reporter gene by STAT3 or STAT3-GFP. Cells were transfected with the reporter gene and genes encoding GFP, STAT3, or STAT3-GFP, and were untreated (open bar) or treated with IFNα (black bar). Fold induction is shown relative to Renilla luciferase transfection controls. **C**) Live cell images of Hep3B cell co-expressing DsRed and STAT3-GFP after serum starvation (time 0) or stimulation with IL-6 (20 ng/ml) for 60 minutes. Nuclear fluorescence (FL) intensity was quantified with time for both fluorescent molecules in the same cell (graph). Results are representative of multiple independent experiments.

Genetically encoded fluorescent proteins allow the study of molecular dynamics and protein-protein interactions in real time without artifacts of fixation necessary for immunofluorescence. As a signaling molecule and transcription factor, STAT3 transmits signals to the nucleus to change gene transcription. In order to evaluate STAT3 nuclear trafficking in a live cell, we tagged STAT3 with the enhanced green fluorescent protein (GFP). We first made certain that the function of STAT3-GFP was comparable to untagged STAT3. Two critical parameters of STAT3-GFP function were evaluated; tyrosine phosphorylation and transcriptional induction. Cells expressing STAT3-GFP or STAT3 were treated with interferon-α (IFNα), and specific tyrosine 705 phosphorylation was evaluated. Results show robust tyrosine phosphorylation of STAT3-GFP similar to the untagged STAT3 (Figure 1A) as has been demonstrated with IL-6 [22]. In addition, the ability of tyrosine-phosphorylated STAT3-GFP to induce gene transcription was evaluated [6], [32]. The influence of STAT3-GFP was tested on expression of a luciferase reporter gene regulated by a STAT binding site [gamma-IFN activated site (GAS)] [33]. Specific induction of reporter gene expression was stimulated in cells similarly by STAT3 and STAT3-GFP in response to IFNα (Figure 1B). Together the results verified the biological action of STAT3-GFP.

Several studies have reported STAT3 accumulates in the nucleus only following tyrosine phosphorylation [31], [34], [35], [36], whereas other reports have demonstrated constitutive nuclear presence of STAT3 independent of tyrosine phosphorylation [13], [22], [37]. To visualize and quantify nuclear accumulation of STAT3-GFP following cytokine stimulation, we analyzed nuclear fluorescence relative to a control fluorescent protein that is not affected by cytokine, DsRed. STAT3-GFP and Ds-Red were co-expressed in Hep3B cells and localization was studied with time-lapse imaging. Nuclear fluorescence intensity was quantified in cells prior to treatment with IL-6 and during one hour of IL-6 stimulation. STAT3-GFP was found to be prominently nuclear both prior to and following IL-6 stimulation (Figure 1C). Accurate tyrosine phosphorylation of STAT3-GFP was verified by Western blot (Figure S1). Our results with live cell imaging demonstrate dominant nuclear accumulation of STAT3-GFP independent of tyrosine phosphorylation.

### Nuclear import rate of STAT3-GFP is unaffected by tyrosine phosphorylation

**Figure 2:**
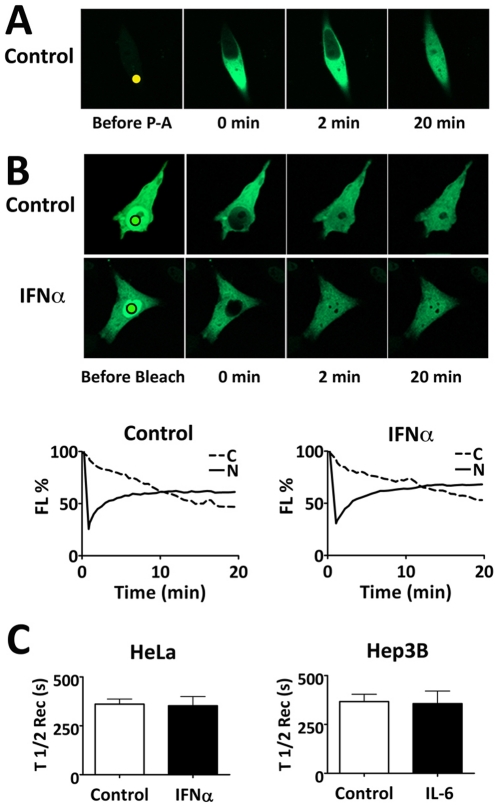
Time-lapse imaging with photoactivation or photobleaching reveals STAT3-GFP continuous nuclear import. **A**) Nuclear import of photoactivatable STAT3 (STAT3-PA-GFP). HeLa cells expressing STAT3-PA-GFP were serum-starved. A region in the cytoplasm (solid dot) was subjected to continuous high-intensity laser to photoactivate the cytoplasmic STAT3-PA-GFP and its nuclear accumulation was evaluated. **B**) Nuclear FRAP experiments were performed with cells expressing STAT3-GFP and serum-starved. A ROI in the nucleus was subjected to high intensity laser (black circle) to bleach nuclear STAT3-GFP. Nuclear fluorescence recovery was evaluated in untreated cells (control) or cells treated with IFNα. Fluorescence intensity was quantified in a ROI in cytoplasm (C) and the nucleus (N) in both untreated (control) and cytokine-treated cells and is shown graphically below. **C**) Multiple experiments with nuclear FRAP of STAT3-GFP were evaluated in HeLa cells treated with IFNα or Hep3B cells treated with IL-6. The half-time (T_1/2_) of nuclear fluorescence recovery was calculated by curve-fitting analysis (GraphPad Prism software) to evaluate STAT3-GFP nuclear import rates in untreated (control) (open bar) or cytokine-treated (black bar) cells.

To study STAT3 movement in living cells, a variant of the GFP molecule was used with very low endogenous fluorescence that increases more than 100-fold following photoactivation [38]. STAT3 was tagged with the photoactivatable-GFP (PA-GFP) and expressed in cells. Cells were serum-starved and a small region of interest (ROI) in the cytoplasm (nanoliter) was subjected to photoactivation with a 2-photon laser system (Figure 2A). The fluorescent signal from STAT3-PA-GFP occurred within seconds of laser stimulation, rapidly distributed within the cytoplasm, and initiated nuclear localization by 2 minutes. A steady-state level of nuclear fluorescence was reached following approximately 20 minutes.

The nuclear import rate of STAT3-GFP was evaluated prior to and following tyrosine phosphorylation using nuclear fluorescence recovery after photobleaching (FRAP) [39] (Figure 2B). A ROI in the nucleus was subjected to laser irradiation to bleach nuclear fluorescence of STAT3-GFP. The subsequent recovery of STAT3-GFP fluorescence in the nucleus was used as a measure of the STAT3-GFP nuclear import rate. Fluorescence recovery in the nucleus was measured by time lapse imaging relative to a ROI in the cytoplasm. Nuclear fluorescence initially increased exponentially and then reached a plateau by 20 minutes as the cytoplasmic fluorescence expectedly decreased. The kinetics of nuclear recovery were found to be similar in either untreated or IFNα-treated cells. To evaluate data from multiple experiments with unphosphorylated and tyrosine phosphorylated STAT3-GFP in HeLa cells or Hep3B cells, curve fitting analyses were performed. Results are presented for the average half-time of recovery for nuclear fluorescence in untreated or cytokine-treated cells from multiple FRAP experiments (Figure 2C). The average half-time of STAT3-GFP nuclear recovery in HeLa or Hep3B cells was similar, in untreated cells or cells stimulated with either IFNα or IL-6, respectively. These results indicate the nuclear import rate is unchanged following tyrosine phosphorylation.

### STAT3-GFP continuous nuclear export

**Figure 3:**
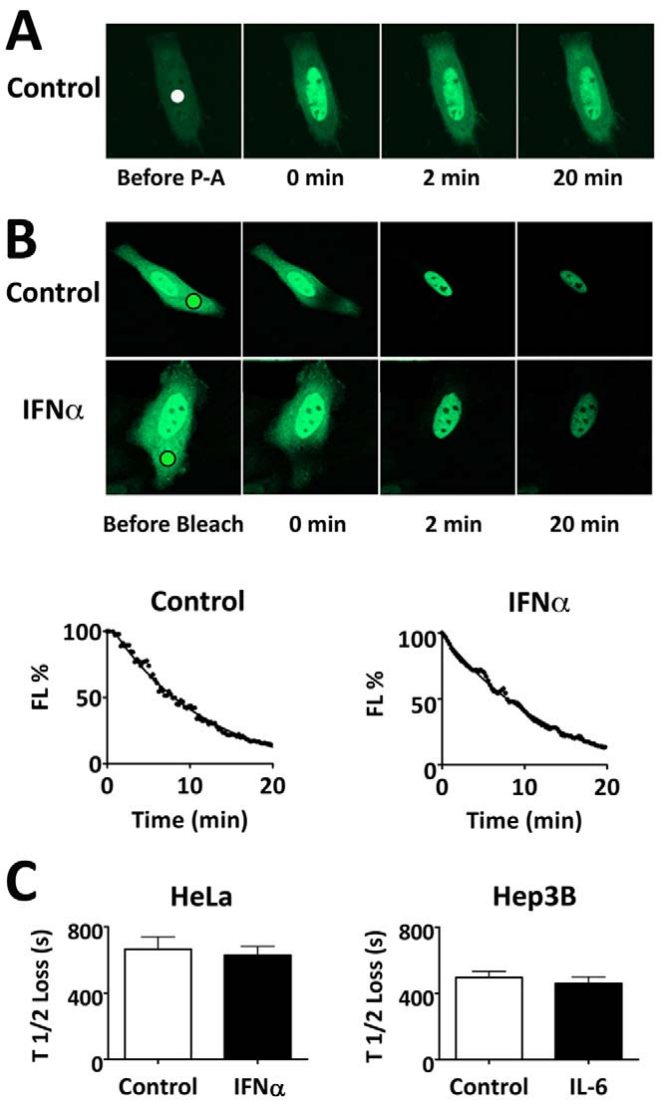
STAT3-GFP nuclear export independent of cytokine stimulation. **A**) Photoactivation of nuclear STAT3-PA-GFP in HeLa cells. A ROI in the nucleus (solid dot) of serum-starved cells was subjected to high intensity laser activation with 2-photon laser microscopy. **B**) Cytosolic FLIP assays were performed in serum-starved HeLa cells expressing STAT3-GFP either untreated (control) or treated with IFNα. A ROI in the cytoplasm (black circle) was subjected to continuous laser bleaching. Time-lapse imaging was used to evaluate loss of fluorescence in the nucleus. Quantitation of fluorescence loss in the nucleus in untreated (control) or IFNα treated cells is shown graphically below. **C**) Kinetics of STAT3-GFP nuclear loss of fluorescence was quantified by curve-fitting analyses of multiple experiments. Results are shown for HeLa cells untreated (control) or treated with IFNα and Hep3B cells untreated or treated with IL-6.

STAT3 cellular localization is dynamic, as it has been shown to enter and exit the nucleus [22]. To evaluate the kinetics of nuclear export we first performed time-lapse imaging with STAT3-PA-GFP (Figure 3A). 2-photon laser microscopy enabled the activation of STAT3-PA-GFP in a ROI limited within the nuclear compartment. The STAT3-PA-GFP rapidly diffused from the ROI to the entire nucleus. Within minutes after photoactivation an increase in cytoplasmic fluorescence was detected, reaching equilibrium by 5–10 minutes.

To assess more accurately the nuclear export rate of STAT3-GFP in untreated or IFNα-treated cells, we used the technique of cytoplasmic fluorescence loss in photobleaching (FLIP) [39](Figure 3B). A small ROI in the cytoplasm was subjected to continuous laser bleaching so that any STAT3-GFP molecules that pass through the path of the laser will be bleached. Within several minutes of cytoplasmic FLIP, the STAT3-GFP in the cytoplasm was completely bleached demonstrating rapid movement throughout the cytoplasm of either untreated or cytokine-stimulated cells. Subsequently, fluorescence in the nucleus decreased indicating STAT3-GFP was exported from the nucleus and bleached by the laser path in the cytoplasm. The kinetics of nuclear export with time was similar in untreated cells or cells treated with IFNα The results support previous findings describing STAT3-GFP nuclear shuttling [22], [27]. Curve fitting analyses were performed with cytoplasmic FLIP to quantify the half-time of fluorescence decrease in the nucleus (export) (Figure 3C). Statistical analyses of multiple experiments were performed with untreated or IFNα-treated HeLa cells or IL-6-treated Hep3B cells. The nuclear export rate of STAT3-GFP was similar in untreated or cytokine-treated cells with no statistically significant differences. A previous report indicated that intra-nuclear mobility of STAT3 increased following cytokine treatment [40], and this might affect export. To determine if mobility of STAT3-GFP within the nucleus before and after cytokine stimulation influenced export, we performed a strip-FRAP within a limited area of the nucleus (Figure S2). In this assay a slower fluorescence recovery indicates slower mobility within the nucleus. Curve fitting analyses indicated a recovery half-time of approximately 0.74 seconds in untreated cells and 1.46 seconds in cytokine-treated cells. Assays with a DNA-binding mutant indicated the slower mobility of tyrosine phosphorylated STAT3-GFP correlated with its ability to bind DNA. Although there was a measurable decrease in intra-nuclear mobility of STAT3-GFP able to bind DNA, this does not appear to significantly influence the rate of STAT3-GFP nuclear export.

### Unphosphorylated STAT3 interaction via the N-terminus

The crystal structure of tyrosine phosphorylated STAT3 bound to DNA has been solved, and it revealed a dimer in which monomer subunits interact via reciprocal SH2 domains and phosphotyrosine residues [32]. This dimer structure is similar to that of the crystal structure of tyrosine phosphorylated STAT1 bound to DNA [41]. Subsequently the crystal structures of unphosphorylated STAT1 and STAT3 were solved. Unphosphorylated STAT1 was also found to exist as a dimer, but with protein interfaces between amino (N)-terminal domains (a.a.1–123) and central core domains (a.a.132–683) in an ‘antiparallel’ orientation in comparison to tyrosine phosphorylated ‘parallel’ orientation [42]. In contrast, the crystal structure of unphosphorylated STAT3 indicated that the core fragment of STAT3 (a.a.127–688) existed primarily as a monomer [43]. Since the core fragment of STAT3 analyzed lacked the N-terminus, it remained possible that full-length unphosphorylated STAT3 formed complexes as suggested previously [37], [44], [45].

**Figure 4:**
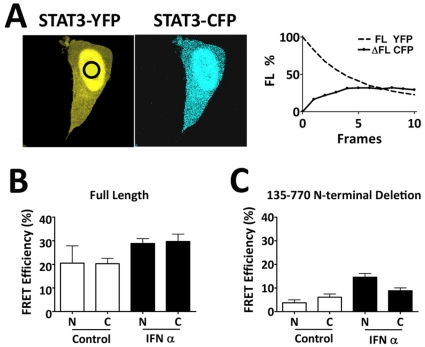
STAT3-STAT3 protein interaction in unphosphorylated and tyrosine phosphorylated states analyzed by FRET. **A**) HeLa cells co-expressing STAT3-YFP and STAT3-CFP were serum starved, fixed, and analyzed for STAT3-STAT3 interactions by FRET. A ROI in the nucleus (black circle) was subjected to laser for gradual photobleaching of STAT3-YFP. Quantification of photobleaching the STAT3-YFP acceptor and the concomitant change in STAT3-CFP fluorescence (FRET) are shown graphically in the right panel. **B**) FRET efficiency was measured between STAT3-YFP and STAT3-CFP in the nucleus (N) or the cytoplasm (C) of multiple serum-starved cells untreated (control) or IFNα-treated cells. **C**) Amino terminal domain of STAT3 is required for unphosphorylated protein interaction. FRET efficiency was measured between STAT3(135–770)-YFP and STAT3(135–770)-CFP in the nucleus or the cytoplasm of multiple untreated (control) cells or IFNα-treated cells.

To evaluate unphosphorylated and tyrosine phosphorylated STAT3 interaction and its impact on nuclear import, we used the technique of Förster resonance energy transfer (FRET) after acceptor photobleaching [46]. FRET occurs between fluorophores that are in close proximity whereby an excited fluorophore transfers energy to a second fluorophore. STAT3-YFP (yellow fluorescent protein) and STAT3-CFP (cyan fluorescent protein) were evaluated as fluorophore pairs co-expressed in HeLa cells (Figure 4A). The technique disrupts energy transfer by selectively photobleaching the YFP group (acceptor), and measuring the fluorescence recovery of the FRET donor group (CFP). The resultant graph depicts the results of FRET analyses between STAT3-YFP and STAT3-CFP and shows an increase in CFP fluorescence after YFP photobleaching. The data indicate FRET interaction and close proximity of unphosphorylated molecules. Curve fitting analyses were performed with data from multiple experiments in cells untreated or treated with IFNα (Figure 4B) [47]. FRET measurements were taken in the nucleus and in the cytoplasm with full length STAT3. STAT3-YFP and STAT3-CFP were found to associate in homomeric complexes either unphosphorylated or following tyrosine phosphorylation in both nuclear and cytoplasmic compartments.

We determined the contribution of the N-terminus of STAT3 (1–134a.a.) to promote interactions of unphosphorylated molecules by measuring FRET between STAT3-YFP and STAT3-CFP molecules lacking the N-terminus (a.a.135–770). The results showed little FRET between unphosphorylated STAT3 deletion mutants, indicating the N-terminus to be critical for interaction between unphosphorylated STAT3 monomers (Figure 4C). The findings support the tenet that oligomerization of unphosphorylated STAT3 molecules is dependent on their N-termini [42]. Our previous studies determined nuclear import to require a region within the coiled coil domain of STAT3 (150–163a.a.), but to be independent of the 1–135a.a. N-terminus [22]. Together with the FRET results it is clear that STAT3 dimerization is not necessary for nuclear import.

### Live cell imaging indicates STAT3-GFP exclusion from mitochondria

**Figure 5:**
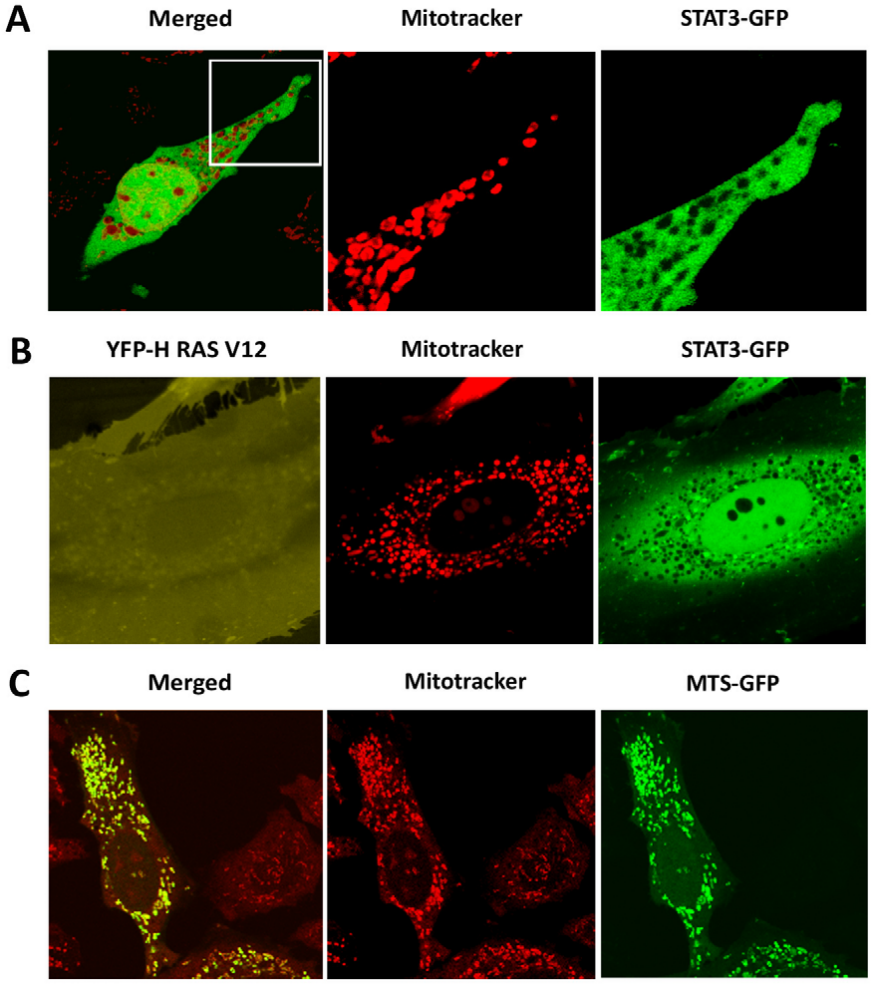
STAT3-GFP is excluded from mitochondria. **A**) HeLa cells expressing STAT3-GFP were stained with MitoTracker Orange and the localization of STAT3-GFP and mitochondria was captured with live cell imaging. **B**) HeLa cells co-expressing STAT3-GFP and YFP-RasV12 were stained with MitoTracker Orange and live cell imaging identified the localization of YFP-RasV12, STAT3-GFP, and mitochondria. **C**) HeLa cells expressing MLS-GFP were stained with MitoTracker Orange and live cell imaging captured the localization of MLS-GFP and mitochondria. Images captured with Zeiss LSM 5 using maximal vertical resolution (<1 µm) and either 40× oil objective or 63× C-Apochromat (water) objective.

Studies using cell fractionation techniques have reported that a portion of STAT3 is localized and functional within mitochondria [14], [15]. To evaluate this possibility we overexpressed STAT3-GFP and used microscopy to visualize the localization of STAT3 in living cells (Figure 5A). MitoTracker Orange, a cell permeable probe, was used to specifically label mitochondria. The use of confocal microscopy with a high vertical resolution setting (<1 µm) indicated STAT3-GFP to be excluded from the interior compartment of the mitochondria. This result is not cell specific and is also shown with Hep3B cells in Figure S3. Since one report described the mitochondrial function of STAT3 with oncogenic H-RasV12 cellular transformation, we evaluated the possible influence of H-RasV12 [15]. We co-expressed YFP-H-RasV12 with STAT3-GFP and stained cells with MitoTracker Orange. However, even in the presence of H-RasV12, STAT3-GFP was not detectable in mitochondria (Figure 5B). To exclude the possibility that the GFP tag was inhibiting mitochondrial import of STAT3, we tested the behavior of GFP tagged with the mitochondrial targeting sequence (MTS) of cytochrome c [48]. The MTS-GFP molecule efficiently accumulated in mitochondria, demonstrating that the GFP tag does not prevent entry into mitochondria (Figure 5C).

### Ran and importin-β1 facilitate STAT3 nuclear import

Active transport is required for large molecules such as STAT3 to enter the nucleus, however the mechanism by which STAT3 gains entrance to the nucleus remained unresolved. Our studies clearly demonstrated the requirement of specific interaction with the adapter importin-α3 for STAT3 nuclear import [22]. Other reports indicated that STAT3 had less discriminate interactions with importin-α adapters [28], [49], or that STAT3 nuclear import was independent of energy or transport carriers [27]. Another group described the requirement for STAT3 association with a GTPase-activating protein (GAP) for Rho GTPases to serve as a chaperone, and the ability of a dominant negative mutant, RacN17, to block STAT3 nuclear import [29]. To address the requirement of a Rac-GAP in STAT3 nuclear import we evaluated the influence of RacN17 (Figure S4). In contrast to their report, we found no evidence for an effect of RacN17 on STAT3 nuclear import in untreated or IFNα-treated cells.

**Figure 6:**
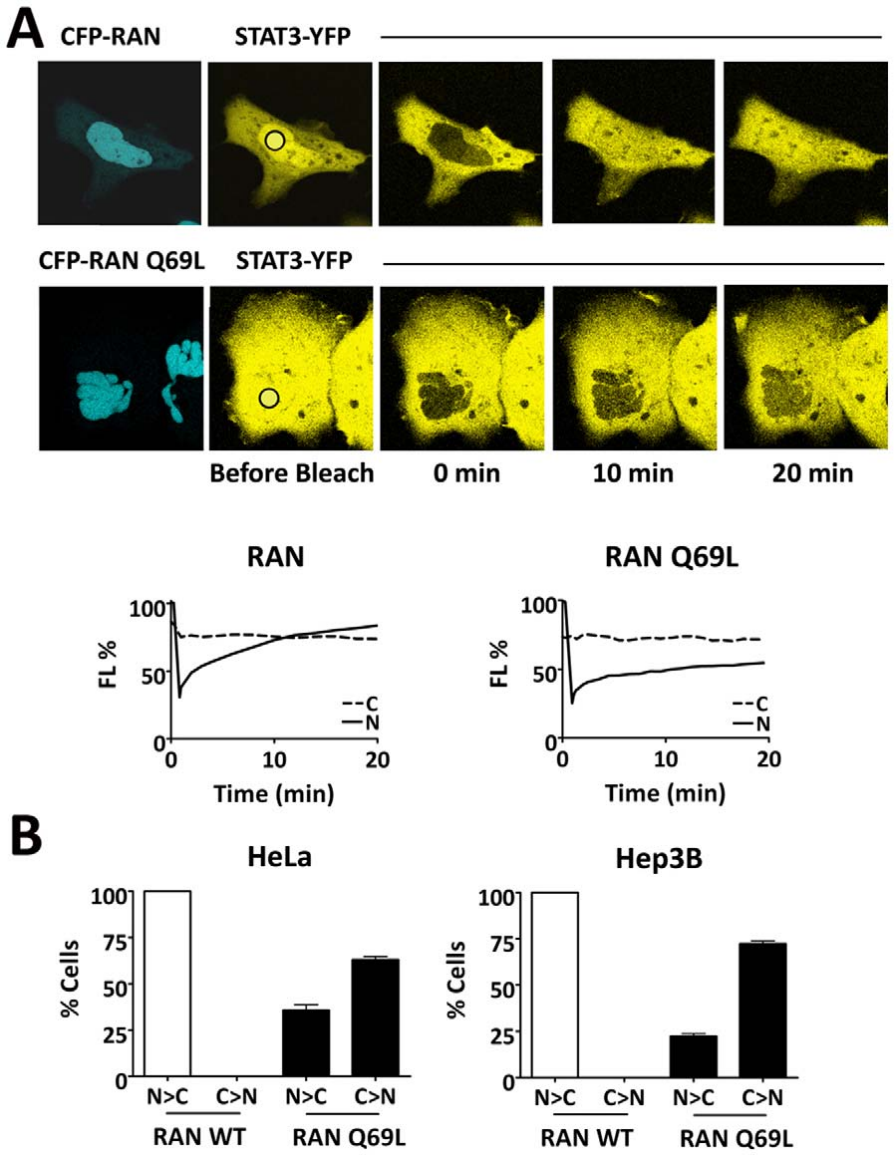
STAT3 nuclear import is dependent on Ran. **A**) Nuclear FRAP experiments were performed to photobleach STAT3-YFP in the nucleus of Hep3B cells co-expressing either CFP-Ran wild type (CFP image top left panel) or CFP-Ran Q69L (CFP image bottom left panel). A ROI in the nucleus was subjected to high intensity laser (black circle) to bleach nuclear STAT3-YFP. YFP fluorescence recovery in the nucleus was followed with time-lapse imaging in cells expressing CFP-Ran wt (top panels) or CFP-Ran Q69L (bottom panels). Quantitation of STAT3-YFP in the nucleus in shown graphically below the microscopic images. Fluorescence was measured in a ROI in the nucleus (N) and a ROI in the cytoplasm (C) in cells expressing wt Ran or Ran Q69L. **B**) Multiple HeLa or Hep3B cells co-expressing STAT3-YFP with either wt Ran or Ran Q69L were evaluated prior to photo-bleaching. The percentage of cells expressing greater nuclear than cytoplasmic fluorescence (N>C) or greater cytoplasmic to nuclear fluorescence (C>N) of STAT3-YFP was measured.

To determine if a classical role of Ran GTPase is involved in the nuclear import of STAT3 by the importin-α/importinβ1 heterodimer, we evaluated the influence of a GTPase mutant of Ran, RanQ69L [24], [50]. RanQ69L remains in a GTP-bound state and is postulated to continually bind to importin-β1 and thereby inhibit the ability of importin-β1 to bind importin-α and transport its cargo to the nucleus. STAT3-YFP was expressed in HeLa cells or Hep3B cells with either wild type (wt) CFP-Ran or CFP-RanQ69L (Figure 6). The nuclear FRAP technique was used with live cell imaging to evaluate the influence of RanQ69L on the rate of STAT3 nuclear import. Cells expressing wt CFP-Ran demonstrated a rate of nuclear fluorescence recovery of STAT3-YFP similar to that seen without Ran overexpression as shown in Figure 2. However, cells expressing CFP-RanQ69L were severely impaired in their ability to import STAT3. Fluorescence in the nucleus only partially recovered by 20 minutes. The effect of RanQ69L on STAT3 nuclear export was evaluated with a cytoplasmic FLIP assay and found to have no demonstrable effect on export (data not shown). The steady state ratio of STAT3-YFP in the nucleus relative to the cytoplasm was quantified in multiple cells expressing CFP-RanQ69L. Although 100% of cells expressing wt Ran had higher levels of STAT3-YFP in the nucleus, in contrast, most of the cells expressing RanQ69L (73%) showed higher levels of STAT3-YFP in the cytoplasm (Figure 6B). Even in the presence of endogenous wt Ran, the RanQ69L impairs STAT3 nuclear import, indicating the critical role of Ran in STAT3 nuclear import.

**Figure 7:**
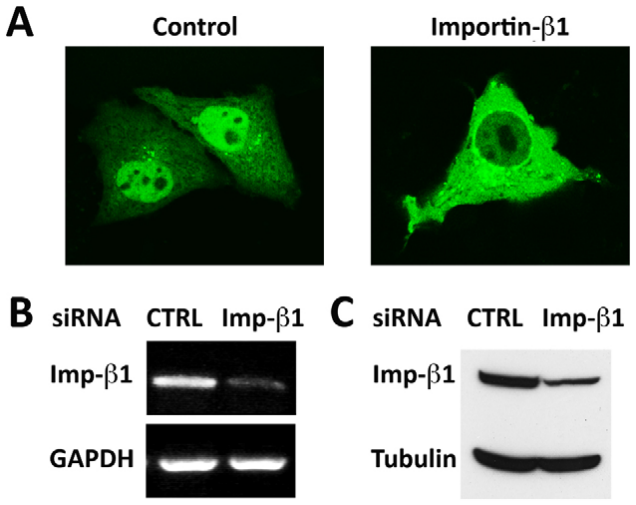
Knockdown of importin-β1 inhibits STAT3-GFP nuclear import. HeLa cells were transfected with vimentin siRNA (control) or importin-β1 siRNA and STAT3-GFP. **A**) Images of STAT3-GFP localization. Four independent experiments indicated 100% of cells expressing control siRNA had prominent nuclear STAT3-GFP. In cultures expressing importin-β1 siRNA, an average of 58% of cells showed a defect in STAT3-GFP nuclear import, with 18% of cells showing the severe impairment indicated in the image. **B**) Effective knockdown of importin-β1 mRNA (Imp-β1) relative to GAPDH mRNA measured in cells expressing importin-β1 siRNA or vimentin siRNA (CTRL) by RT-PCR. Average knockdown of importin-β1 mRNA from four experiments was approximately 56% measured with Image J software. **C**) Knockdown of importin-β1 protein relative to tubulin was approximately 50% evaluated by Western blot.

The involvement of Ran-GTP in the regulation of STAT3 nuclear import along with our previous finding of the role of importin-α3 suggested that importin-β1 would play a critical role. To directly evaluate the requirement of importin-β1 for STAT3 nuclear import we tested the effect of RNA interference. siRNA duplexes corresponding to a control gene (vimentin) or to importin-β1 were transfected into cells to decrease mRNA and protein levels. Subsequently STAT3-GFP was transfected into cells and evaluated for cellular localization (Figure 7). There was a significant inhibition of STAT3-GFP nuclear accumulation in approximately 58% of the cells treated with importin-β1 siRNA whereas there was no effect of control siRNA. The results strongly suggest that STAT3 nuclear import is mediated by the importin-α-importin-β1-Ran transport system.

## Discussion

STAT3 is activated by tyrosine phosphorylation in response to a variety of cytokines and growth factor receptor tyrosine kinases. For this reason it is not surprising that STAT3 serves critical functions in diverse physiological responses. However, persistent activity of STAT3 in response to unregulated hormone signaling or oncogenic tyrosine kinases leads to the induction of genes that positively control proliferation in cancer, tumor cell invasion, or chronic inflammation that can progress into life-long autoimmune diseases [4], [6], [51], [52], [53], [54], [55], [56], [57], [58]. These facts point to the need to understand the mechanisms that regulate STAT3 function in order to develop strategies to block its action in disease.

To transmit extracellular signals to the nucleus, STAT3 needs the ability to shuttle between cytoplasmic and nuclear compartments. We have provided evidence with live cell imaging that the nuclear import of STAT3 and nuclear export of STAT3 occurs continually independent of its state of tyrosine phosphorylation. In addition, the rate of import and export does not change following phosphorylation. Our studies are not dependent on a fixation process for immunofluorescence that can perturb cellular architecture or can be influenced by cross-reactive antibodies. The results are consistent with previous evidence identifying a nuclear import signal residing within the coiled coil domain of STAT3 [22], [36]. Judging from the crystal structures of STAT3, the coiled coil domain is accessible to importins both prior to and following tyrosine phosphorylation [32], [43].

We have provided evidence that unphosphorylated STAT3 forms dimers or oligomers by homomeric interaction between their N-termini (1–135a.a.). The FRET technique with photobleaching revealed STAT3 interactions in both tyrosine phosphorylated and unphosphorylated forms. Although the crystal structure of unphosphorylated STAT3 core fragment was solved as a monomer, this core fragment lacked the N-terminus. Our results support the concept that the structures of unphosphorylated and tyrosine phosphorylated STAT3 may be similar to that of STAT1. The tyrosine phosphorylated dimers associate via the SH2 domain and phosphotyrosine in a ‘parallel’ conformation, whereas the unphosphorylated dimers associate via N-termini in an ‘anti-parallel’ conformation [41], [42], [59]. There was an overall lower level of oligomerization detected by FRET following tyrosine phosphorylation of the N-terminal deletion that may be due to transition kinetics from unphosphorylated monomer to phosphorylated dimer compared to unphosphorylated dimer to phosphorylated dimer. It may also reflect a lack of tetramer formation between phosphorylated dimers [60]. In any case, the nuclear import of STAT3 is independent of its phosphorylation and its dimerization.

The spatial and temporal localization of a protein within the cell can dictate its biological effects. Two studies have used cell fractionation to report STAT3 localization within mitochondria to regulate oxidative phosphorylation [14], [15]. This technique uses cell disruption and differential centrifugation to enrich for organelles, membranes, or soluble molecules. However, caveats include incomplete cell lysis, contamination with nuclei, and artificial protein association after cell lysis [61]. A recent study used proteomic methods to evaluate the stoichiometry of STAT3 and mitochondrial proteins and concluded there was not sufficient STAT3 in the cell to directly influence mitochondrial complexes I/II [62]. To visually evaluate the localization of STAT3 in mitochondria, we used live cell imaging with laser scanning confocal microscopy and a high vertical resolution. Mitochondria were stained with MitoTracker Orange, a cell permeable dye, and STAT3-GFP was overexpressed in order to detect possible mitochondrial localization. We did not detect STAT3-GFP in the mitochondria. Even with the co-expression of H-RasV12, which was reported to require mitochondrial STAT3 to transform cells, STAT3-GFP appeared to be excluded from mitochondria. We cannot eliminate the possibility that STAT3 interacts with the outer membrane of mitochondria, or that a very small amount of STAT3 enters mitochondria below the detection level. However, the results indicate that the influence of STAT3 on mitochondrial function may be mediated by regulation of gene expression in the nucleus.

The mechanism by which STAT3 is imported to the nucleus has been proposed to occur independent of active transport and importins, and alternatively to require association with a Rac-GAP [27], [29]. Our studies with a dominant negative Rac did not support the involvement of a Rac-GAP in STAT3 nuclear import. In addition, we previously showed that importin-α3 direct binding to STAT3 plays a critical role in its import [22]. To more extensively investigate the mechanism of STAT3 nuclear import, we evaluated the contribution of Ran and importinβ1. Ran GTPase regulates the translocation of proteins through the nuclear pore by influencing the ability of importin-β1 to bind importin-α [18], [19], [20], [21]. Ran in a GTP-bound state binds to importin-β1, triggering disassembly of complex association with importinαand cargo. We used the Ran Q69L mutant that is continually in a GTP-bound state to determine its influence on STAT3-GFP nuclear import. Even in the presence of endogenous wt Ran, Ran Q69L significantly inhibited STAT3-GFP import, indicating an active transport mechanism dependent on Ran. Furthermore we used RNA interference to reduce the cellular levels of importin-β1 RNA and found nuclear import of STAT3-GFP was inhibited. These data together demonstrate that STAT3 is imported into the nucleus by importin-α/importin-β1-Ran-mediated active transport.

In conclusion, our live cell imaging studies clearly show that STAT3 is continuously imported to the nucleus and exported independent of tyrosine phosphorylation, and nuclear import is mediated by the importin-α/importin-β1-Ran system. This knowledge of the molecular interactions that mediate STAT3 nuclear trafficking is critical to provide a basis to develop strategies to block its action in cancer and autoimmunity.

## Materials and Methods

### Cell Culture, Transfection, and Cytokine Treatment

HeLa, Hep3B, and 293FT cells (ATCC) were cultured in DMEM with 10% fetal bovine serum, 1 mM L-glutamine, and 1% v/v penicillin/streptomycin. For microscopy, cells were seeded in glass bottom tissue culture dishes (Mattek Corp.) or on glass coverslips. Transfections were performed with TransIT-LT1 reagent (Mirus Bio LLC). Cells were cultured in serum-free media for 12 hours prior to experiments. Cells were treated with 20 ng/ml IL-6 (BioSource International), IFNα 1000 units/ml (gift from Roche, Nutley, NJ), or TNF (Invitrogen PHC3015).

### Plasmids

STAT3 (gift of James Darnell, Jr., The Rockefeller University) was cloned into the vectors pEGFP-N1 (STAT3-GFP), pEYFP-N1 (STAT3-YFP), and pECFP-N1 (STAT3-CPF) (Clontech). STAT3 DNA-binding mutant (VVV) was cloned into pEGFP-N1 [63]. The N-terminus deletion mutant construct (STAT3-135-770-GFP) was generated previously [22]. The vector encoding photoactivatable GFP (PA-GFP) was a gift of Jennifer Lippincott-Schwartz (NIH) and used to generate STAT3-PA-GFP [38]. pDsRed-N1 was obtained from Clontech. Human Ran and mutant Ran Q69L were gifts from Colin Dingwall (Kings College, London) and were sub-cloned in pECFP-C1 (CFP-Ran and CFP-Ran Q69L) [64]. T7-tagged-Rac1-N17 and Rac1 were gifts of Linda Van Aelst (Cold Spring Harbor Laboratory). Harvey Ras-V12 was a gift of Dafna Bar-Sagi (New York University) [65] and was subcloned into pEYFP-C1 (Clontech). The luciferase reporter gene regulated by the STAT-responsive gamma IFN activated site has been described previously [58]. The NF-κB-responsive luciferase reporter gene was obtained from Stratagene, and the Renilla luciferase gene was obtained from Promega.

### RNA interference

Cells were treated with small interfering RNA (siRNA) corresponding to importin-β1 or vimentin control (Qiagen) as described [66]. Briefly, siRNAs were transfected into cells using X-tremeGENE siRNA transfection reagent (Roche) and 24 hours later the cells were transfected with STAT3-GFP plasmid. STAT3-GFP fluorescence was evaluated by confocal microscopy 48 hours after siRNA transfection. RT-PCR was used to quantify endogenous importin-β1 mRNA and GAPDH mRNA as described previously [66].

### Antibodies

Western blots were performed as described previously using the following antibodies: anti-phospho-tyrosine (p705) STAT3 (SantaCruz, Sc-8059/clone B7), anti-STAT3 (Santa Cruz sc-482), anti-importin-β1 (Santa Cruz H-7 sc-137016), anti-tubulin (Sigma B5-1-2), anti-rabbit (Alexa-labeled Invitrogen A21109), and anti-mouse HRP (Amersham Biosciences, NA931V) [22]. Antibodies for immunofluorescence were anti-T7 (Novagen) and rhodamine-conjugated anti-mouse (Jackson Laboratory).

### Confocal Microscopy

Live cell imaging was performed using a Zeiss LSM 510 META NLO Two-Photon Laser Scanning Confocal Microscope System and cell chamber system with 37°C temperature control (Temperature Control 37-2, and Heating Insert P from Zeiss) and CO_2_ control (CTI Controller 3700, and Incubator S from Zeiss) [66], [67]. Images were captured and analyzed using the imaging software Zeiss LSM 510 Meta version 3.2 and Image J. Images are presented using Adobe Photoshop graphic software. GraphPad Prism software was used for curve-fitting analyses. Fluorescence recovery after photobleaching (FRAP) with STAT3-GFP or STAT3-YFP was performed by bleaching a region of interest (ROI) in the nucleus at 100% power of an argon laser (488 nm for GFP, 514 nm for YFP) for a duration of time ranging from 60 to 120 seconds. Fluorescence loss in photobleaching (FLIP) was performed by bleaching a ROI in the cytoplasm, repeatedly every 12 seconds at maximum laser intensity. Photoactivation experiments were performed using the two photon laser system Chameleon XR Laser System, with the following settings: 800 nm laser, laser power of 10–20%, and 50–100 laser continual iterations. Förster resonance energy transfer (FRET) experiments were performed using the method of “FRET after acceptor photobleaching” using CFP and YFP fluorophore pairs [47], [68]. HeLa cells were grown on glass coverslips and transfected with DNA ratio 1∶3 between the CFP constructs and YFP constructs. 2 days post transfection the cells were washed twice in cold PBS and fixed with 4% paraformaldehyde. The coverslips were washed in PBS and mounted onto microscope glass slides using mounting media Vectashield (Vector Laboratories) and sealed. STAT3-YFP was excited and bleached and the resultant energy transfer to STAT3-CFP was quantified. The microscope settings were as follows: objective lens 63× C-Apochromat, laser 458 nm for CFP excitation, laser 514 nm for YFP excitation and bleaching, filter lambda mode for CFP emission 458–510 nm, and filter lambda mode for YFP emission 530–630 nm. The FRET efficiencies were calculated by curve fitting analysis of CFP fluorescence versus YFP fluorescence using the mathematical approach of Amiri et al. [47]. For STAT3-GFP mitochondrial localization, live cells were treated with 50 nM of the cell permeable MitoTracker Orange fluorescence dye (Molecular Probes, Invitrogen) for 1 hour and subsequently washed with media. Live cell imaging was performed using the following microscope settings: objective lens 63× C-Apochromat, laser 488 nm for GFP excitation, laser 543 nm for mitotracker orange, and laser 514 nm for YFP, filter BP 500–550 IR for GFP emission, filter BP 565–615 IR for MitoTracker Orange emission and filter BP 535–590 for YFP emission. Different fluorescence channel images were acquired sequentially. In order to achieve a satisfactory vertical resolution (<1 µm) the imaging experiments were performed with limited pinhole size of 100–130 µm.

## Supporting Information

Figure S1 **Accurate tyrosine phosphorylation of endogenous STAT3 and STAT3-GFP in HeLa and Hep3B cells in response to cytokines.** Cells were transiently transfected with STAT3-GFP and serum-starved overnight. HeLa cells were treated with 1000 U/ml IFNα for one hour and Hep3B cells were treated with 20 ng/ml IL-6 for one hour. Endogenous STAT3 (ENDO) and STAT3-GFP were detected by Western blot with antibodies to STAT3 (bottom panel) or to tyrosine phosphorylated STAT3 (pY-STAT3) (top panel).
(TIF)

Figure S2 **STAT3 intra-nuclear mobility reduced with DNA binding.** Nuclear FRAP experiments were performed in HeLa cells expressing STAT3-GFP or a mutant of STAT3 that cannot bind DNA (STAT3-VVV-GFP) with or without IFNα stimulation. A limited ROI strip area (31 µm^2^) was selected for photobleaching, and kinetics of fluorescence recovery into this area was quantified. **A**) Time-lapse imaging of STAT3-GFP to evaluate intranuclear mobility. **B**) Graphic quantitation of multiple experimental results with STAT3-GFP in serum-starved cells untreated (control, solid line) or IFNα-treated (dashed line). **C**) The half-time of fluorescence recovery in seconds was calculated from multiple experiments in serum starved cells untreated (control, open bars) or IFNα-treated (black bars) for wild type STAT3-GFP (0.74 sec untreated; 1.46 sec treated) or the DNA binding mutant STAT3-VVV-GFP (0.75 sec untreated; 0.93 sec treated).
(TIF)

Figure S3 **STAT3-GFP exclusion from mitochondria in Hep3B cells.** Hep3B cells expressing STAT3-GFP were treated with MitoTracker Orange, a cell permeable dye for mitochondria. Live cell imaging was used to detect the localization of STAT3-GFP and mitochondria. Images captured with Zeiss LSM 5 using maximal vertical resolution (<1 µm) with 40× oil objective or the 63× C-Apochromat (water) objective.
(TIF)

Figure S4 **STAT3 nuclear import independent of Rac1.** **A**) HeLa cells co-expressing STAT3-GFP and T7-Rac-N17 were serum-starved and untreated (control) or stimulated with IFNα Nuclear prominence of STAT3-GFP in cells expressing RacN17 is shown in left panels. Immunofluorescence of T7-RacN17 is shown in center panels with anti-T7 antibody. Merged images clearly indicate nuclear localization of STAT3-GFP in cells expressing Rac1-N17. **B**) The dominant negative action of Rac1-N17 was demonstrated by its ability to block Rac1-mediated activation of NF-κB in a dose-reponsive manner [69]. HeLa cells were co-transfected with NF-κB responsive luciferase reporter, Renilla luciferase, and T7-tagged empty vector, Rac1, or Rac1-N17 constructs. Ratio of plasmid DNA corresponding to Rac1 and Rac1-N17 was 1∶1 or 1∶3. Cells were serum starved overnight (open bars) followed by TNFα (10 ng/ml) treatment for 6 hours (black bars). Fold induction is shown relative to Renilla luciferase transfection controls.
(TIF)
