# Supplementary figures and images for: Dynamics of the STAT3 Transcription Factor: Nuclear Import Dependent on Ran and Importin-β1
Source: PLoS One. 2011 May 19;6(5):e20188. doi: 10.1371/journal.pone.0020188 (PMC3098288; doi:10.1371/journal.pone.0020188)

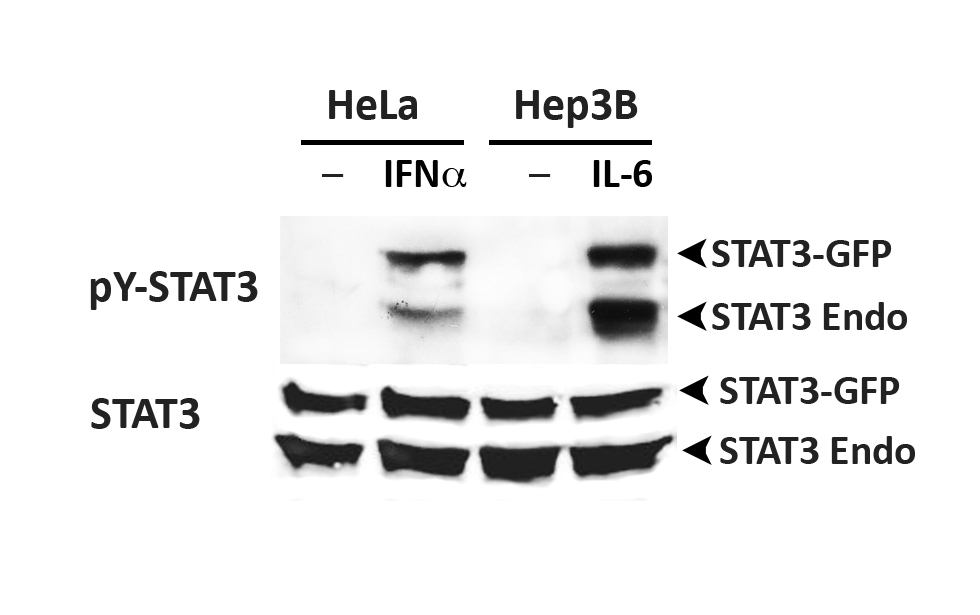

Supplement: Figure S1 — Accurate tyrosine phosphorylation of endogenous STAT3 and STAT3-GFP in HeLa and Hep3B cells in response to cytokines. Cells were transiently transfected with STAT3-GFP and serum-starved overnight. HeLa cells were treated with 1000 U/ml IFNα for one hour and Hep3B cells were treated with 20 ng/ml IL-6 for one hour. Endogenous STAT3 (ENDO) and STAT3-GFP were detected by Western blot with antibodies to STAT3 (bottom panel) or to tyrosine phosphorylated STAT3 (pY-STAT3) (top panel). (TIF) [file pone.0020188.s001.tif]
